# Relationship between preterm, low birth weight and early childhood caries: a meta-analysis of the case–control and cross-sectional study

**DOI:** 10.1042/BSR20200870

**Published:** 2020-08-11

**Authors:** Linan Shi, Jinhai Jia, Chunnian Li, Caiyun Zhao, Ting Li, Hong Shi, Xiaolin Zhang

**Affiliations:** 1 Department of Pediatric Dentistry, School and Hospital of Stomatology Hebei Key Laboratory of Stomatology, Hebei Medical University, Shijiazhuang, 050017, China; 2 Outpatient Clinic, Hebei Medical University, Shijiazhuang 050017, China; 3 Department of Endodontics, School and Hospital of Stomatology Hebei Key Laboratory of Stomatology, Hebei Medical University, Shijiazhuang 050017, China; 4 Department of Pathology, School and Hospital of Stomatology Hebei Key Laboratory of Stomatology, Hebei Medical University, Shijiazhuang 050017, China; 5 Department of Epidemiology and Statistics, School of Public Health, Hebei Province Key Laboratory of Environment and Human Health, Hebei Medical University, Shijiazhuang, Hebei 050017, China

**Keywords:** early childhood caries, low birth weight, meta analysis, preterm

## Abstract

Early childhood caries (ECC) is one of the most prevalent chronic infectious diseases in children. The effective prevention and treatment are heavy burdens and study hotspots for pediatric dentists. Many studies had investigated the relationship between preterm, low birth weight (LBW) and ECC; however, the results were inconsistent. The present study was conducted with an evidence-based study to figure out the relationship between preterm, LBW and ECC for the first time. After searching the database, case–control and cross-sectional studies relevant to the relationship between preterm, LBW and ECC up to December 2019 were included. The data about odds ratio (OR) and 95% confidence interval (95% CI) were extracted and calculated with STATA 14.0 Software. A total of 22 studies were included in this meta-analysis, 9 studies of which did not only explore the relationship between ECC with preterm, but also study the relationship between ECC and LBW, 7 studies of which explored the relationship between preterm and ECC, and 6 studies of which studied the relationship between LBW and ECC. The meta-analysis results showed that the preterm increased the risk of ECC (OR = 1.59, 95% CI: 1.36–1.87) significantly. There was no difference between LBW and normal birth weight in the incidence of ECC (OR = 1.12, 95% CI: 0.94–1.33). The meta-analysis results of adjustment OR about LBW were similar to the crude OR (OR = 1.05, 95% CI: 0.71–1.57). This meta-analysis indicated that preterm increased the risk of ECC significantly; however, LBW was not a risk factor for ECC.

## Introduction

The definition of Early Childhood Caries (ECC) was proposed by American Academy of Pediatric Dentistry (AAPD) in 1999: the presence of one or more cavitated or non-cavitated lesion, missing or filled tooth/teeth due to caries in any primary tooth in a child of 71 months or younger [1]. ECC is one of the most prevalent chronic infectious diseases in children, especially in the developing countries. According to the Fourth National Oral Health Epidemiology Survey of China, the prevalence of ECC occurred in 5-year-old children reached 71.9% [2], which also showed an upward trend. ECC had negative influences on children’s physical and mental development. Currently, the most effective measures for preventing ECC were some behavior managements against the etiology and risk factors, such as controlling the intake of sugar-sweetened foods and increasing tooth brush frequencies [3,4]. Meanwhile, whether infants birth condition and maternal health status during pregnancy influenced the caries experience of children were always the study hotspots, and the mechanisms of the relation were still uncertain [5,6]. Previous studies suggested that adverse birth outcomes, such as preterm or low birth weight (LBW), were the risk factors for ECC, but there were other studies indicating the opposite results [7,8].

The World Health Organization (WHO) defined preterm birth as the birth of an infant prior to 37 weeks of gestation [9]. The incidence of preterm had reached 1/10 worldwide, even 1/3 in some developing countries [10]. The mechanisms and causes of preterm were still unknown. Previous studies suggested that low socioeconomic and educational status, maternal malnutrition or infection during pregnancy might result in infants small for gestation [11]. In 1962, Rosenzweig reported the enamel hypoplasia (EHP) and enamel caries in the primary dentition of premature for the first time [12]. Since then, numerous of studies were focused on the dental structures of premature or LBW infants and many epidemiologic studies were conducted to investigate the incidence of ECC in premature or LBW infants. Many studies suggested preterm children were more prone to get ECC because of the disturbances in growth and development of primary dentition, the difficulties in keeping oral hygiene and the enamel mineralization [13–15]. By contrast, numerous of studies concluded that preterm was not a risk factor for ECC due to the delayed teeth eruption, and preterm infants experienced more follow-up and acquired more detailed knowledge to keep oral health [16].

LBW is defined as the newborn babies weighing less than 2500 g at birth [9]. Either preterm or intrauterine growth restriction (IUGR) retarded the weight growth [9]. In 2012, the prevalence of LBW was 8% in America, two-thirds of which were preterm infants. Seow found that the enamel of deciduous teeth developed at the 14th week after ingravidation and lasted throughout pregnancy until the first year after birth [17]. Nelson et al. reported that EHP was closely associated with LBW, especially very low birth weight (VLBW), as deficiency of vitamin D, calcium and phosphate which were difficult to be transported to fetus when pregnant women were experienced maternal malnutrition and metabolic disturbance [18]. The development of EHP and the colonization of cariogenic bacteria predisposed them to ECC [15]. The conclusion was inconsistent with many studies that indicated that there was no relationship between LBW and ECC.

This meta-analysis was conducted based on uniform diagnosing criteria and collected all the studies that met the inclusion criteria, systematically assessed the relationship between preterm, LBW and ECC. The results of this meta-analysis provided scientific evidence for the etiology and prevention of ECC.

## Methods

### Focused question

This meta-analysis was conducted according to the Preferred Reporting Items for Systematic Reviews and Meta-Analyses (PRIMSA). The question was whether preterm and LBW infants compared with the children under normal birth conditions were susceptible to ECC, which was posed in accordance with the Participants, Interference, Control, Outcome, Study Design(PICOS) principle.

### Search strategy

The following databases were searched (up to December 2019): PubMed, Wiley, Cochrane Library, Science Direct, China National Knowledge Infrastructure (CNKI), VIP Database for Chinese Technical Periodicals (VIP) and Wanfang. The relevant references of all retrieved articles were included. The complete search strategy was performed using the following Boolean phrases: (preterm OR premature OR prematurity OR low birth weight) AND (caries OR early childhood caries) AND (children OR preschool children).

### Inclusion criteria

Two reviewers identified and selected potentially relevant studies independently by reading titles, abstracts and full texts. The studies were selected based on the following inclusion criteria: (1) the subjects were younger than or equal to 6 years old; (2) premature or LBW were identified as the exposure factors in the literature; (3) the methods of the study were epidemiological studies (case–control study, cross-sectional study); (4) the criteria for diagnosing whether one child had caries was one or more primary tooth/teeth had present tissue loss and cavitation, missing or had been filled because of caries, which was in accordance with the standard proposed by the WHO; (5) the outcome indicators were odds ratio (OR) or adjustment OR and 95% confidence interval (95% CI).

### Exclusion criteria

The reviewers read the full-text publications based on the following exclusion criteria: (1) the examiners did not carry out the test or training of the reliability of the results; (2) the study language was not English or Chinese; (3) repeated study or there was no sufficient data to calculate OR and 95% CI; (4) Gray literature.

### Data extraction

Data extraction was conducted by two independent reviewers; disagreement would be resolved by consensus. The corresponding risk estimations (including ORs), which were adjusted or not for the maximum number of confounding variables with corresponding 95% CIs, were extracted. The following information was extracted from each included study: the first author, the year of publication, the study method, the number of patients and the age range of patients.

### Quality assessment

The reviewers assessed the quality of all the included studies based on the JBI scale for cross-sectional studies, and the score reached 70% of full mark was a low bias risk. The Newcastle–Ottawa Scale (NOS) was adopted for the quality evaluation of case–control studies. Studies with scores of 0–3, 4–6 and 7–9 were considered as low, moderate and high quality, respectively.

### Statistical analysis

The OR was used as the common measure of associated across studies. Heterogeneity across studies was assessed using the Cochrane Q Statistic(significance level at *P*<0.10) and the *I^2^* statistic. The heterogeneity was considered as statistically insignificant if *P*>0.10 and *I^2^*≤50%, and then the Mantel–Haenszel fixed-effect model was used for calculating pooled OR among studies. Otherwise, the DerSimonian and Laird random-effect model was used for combining the results [19]. Sensitivity analysis was performed to detect the effects of an individual study on the pooled results by omitting one study in each turn. Potential publication bias was detected by Peters’ test. The data were dealt with STATA Version 14.0 Software (STATA Corporation, College Station, TX, U.S.A.).

## Results

### Literature search and study characteristics

**Figure 1:**
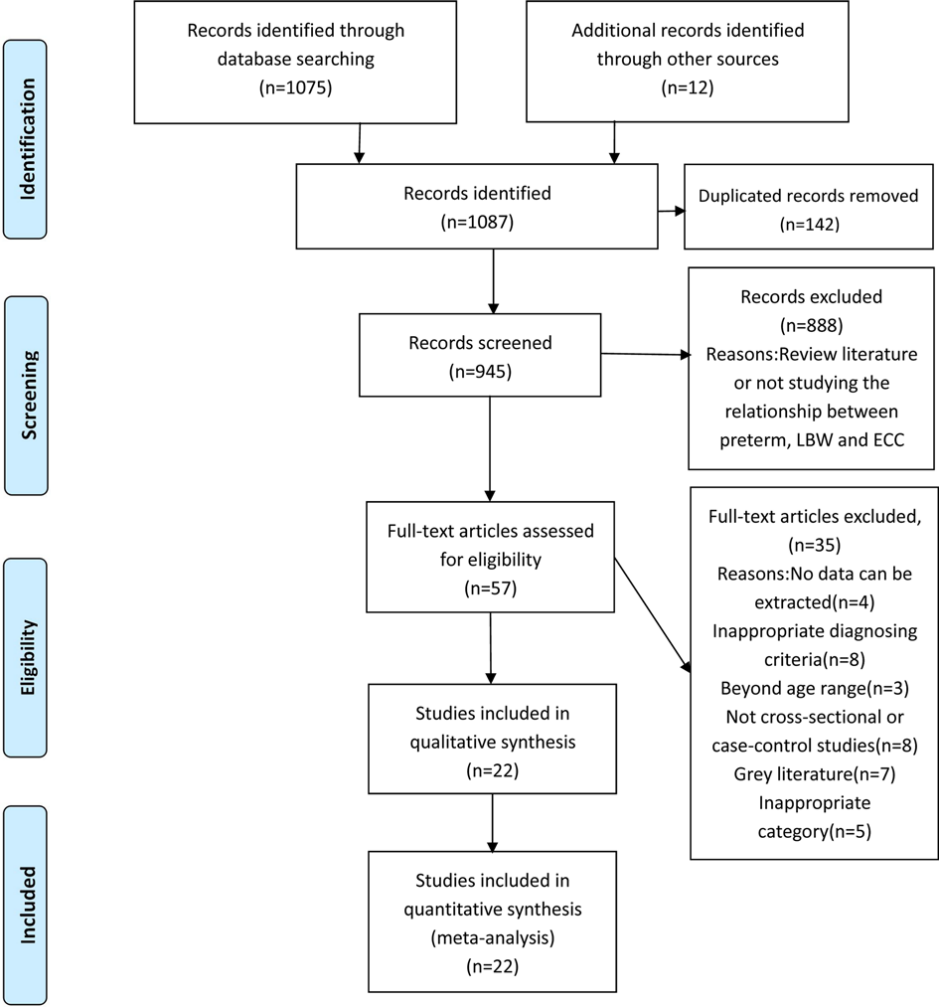
PRISMA 2009 Flow Diagram

The flow diagram of the study selection process is shown in Figure 1. A total of 1087 articles were identified according to the search strategy, 142 articles of which were excluded because of duplication. By screening of titles or abstracts further, 888 articles were excluded as they were review literatures or not studying the relationship between preterm, LBW and the incidence of ECC. After reading the full text of the remained articles, 35 articles were excluded because no data could be extracted or they were not case–control or cross-sectional studies. Overall, 22 studies conforming to the inclusion criteria were included in the meta-analysis [15,20–40]. Nine of these twenty-two studies did not only explore the relationship between ECC and preterm, but also studied the relationship between ECC and LBW, and seven studies explored the relationship between preterm and ECC, and six studies investigated the relationship between LBW and ECC. Table 1 summarized the main characteristics of the selected studies for analysis. Seven of these twenty-two studies were case–control studies and fifteen studies were cross-sectional studies. The population size of per study ranged from 128 to 5275 and totally 25,166 participants were included in the systematic meta-analysis. All these included 15 cross-sectional studies and 2 case–control studies were with high quality and the other case–control studies were scored 5 or 6 with moderate quality.

**Table 1:** The characteristics of the including studies

Study ID	*n*	Case group (ECC/Health)		Control group (ECC/Health)		Age range	Study method	Quality score
		Preterm group	LBW group	Preterm group	LBW group			
Sridevi, 2018	690	142/99	40/39	203/246	305/306	3–6 y	Case–control	8
Hou, 2008	290	16/12	2/11	89/172	104/173	2–4 y	Case–control	6
Chen, 2015	3243	103/100	51/57	1307/1735	1357/1778	3–6 y	Case–control	6
Rajshekar, 2011	500	120/130	120/130	97/153	97/153	1–6 y	Case–control	7
Schuler, 2017	128	11/53	11/53	7/57	7/57	3–4 y	Case–control	6
Miao, 2011	588	13/12	6/15	267/296	274/293	3 y	Case–control	5
Saravia, 2007	3189	92/183	55/137	592/2322	624/2373	2–6 y	Cross-sectional	18
Zhou, 2011	394	29/88	1/7	80/197	108/278	2 y	Cross-sectional	17
Li, 1996	1290	74/20	38/7	983/213	1005/225	3–5 y	Cross-sectional	18
Miao, 2015	496	17/7		309/163		3–5 y	Case–control	6
Yao, 2018	737	51/46		233/407		3–6 y	Cross-sectional	15
Campus, 2008	5275	136/397		985/3757		4 y	Cross-sectional	18
Ma, 2016	598	40/17		272/269		3–6 y	Cross-sectional	15
Zhao, 2010	1875	93/28		982/772		3–6 y	Cross-sectional	15
Yang, 2014	578	28/10		337/203		3-5 y	Cross-sectional	15
Zhen, 2016	1408	245/217		341/605		3–6 y	Cross-sectional	16
Masumo, 2014	750		23/63		115/549	6–36 m	Cross-sectional	18
Olatosi, 2014	239		15/51		38/135	6–71 m	Cross-sectional	17
Gopal, 2016	477		46/115		84/232	3–6 y	Cross-sectional	17
Li, 2017	343		4/5		194/140	5 y	Cross-sectional	16
Prakash, 2012	1500		135/364		278/723	8–48 m	Cross-sectional	16
Correa-Faria, 2013	578		27/30		280/241	3–5 y	Cross-sectional	18

### Meta-analysis results

**Figure 2:**
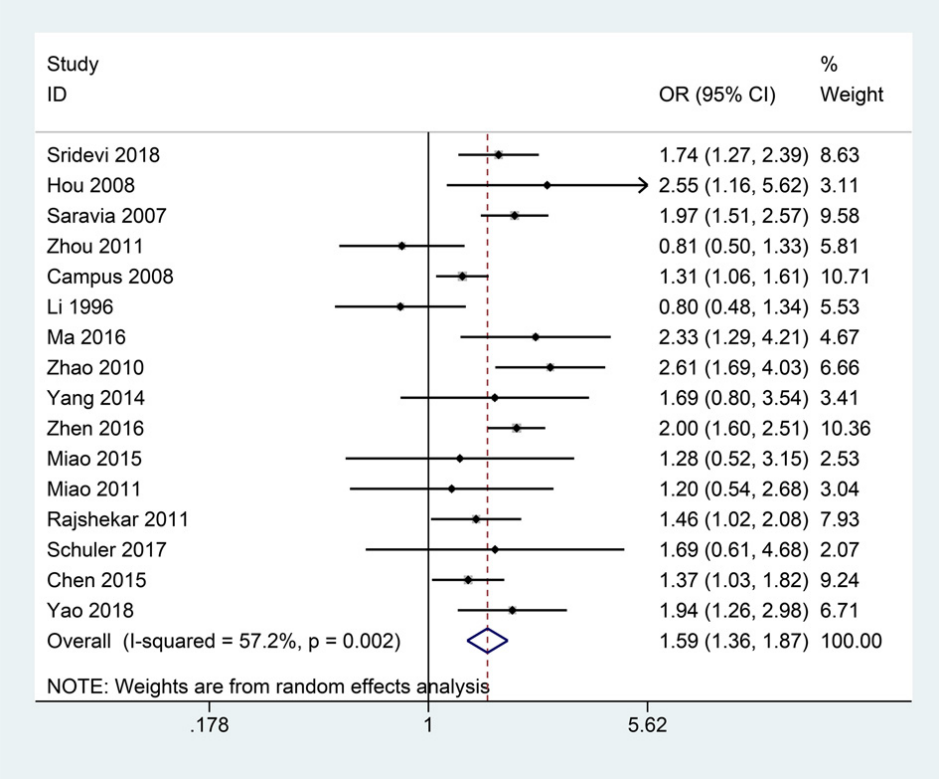
Forest plots showed the relationship between preterm and ECC

Figure 2 shows that forest plot presented the relation between preterm and ECC. Heterogeneity across studies was *P*_heterogeneity_<0.01, *I^2^*= 57.2%. The meta-analysis results using a random-effect model suggested an increased risk of ECC in premature compared with full-term children (OR = 1.59, 95% CI: 1.36–.87, *P*<0.01).

**Figure 3:**
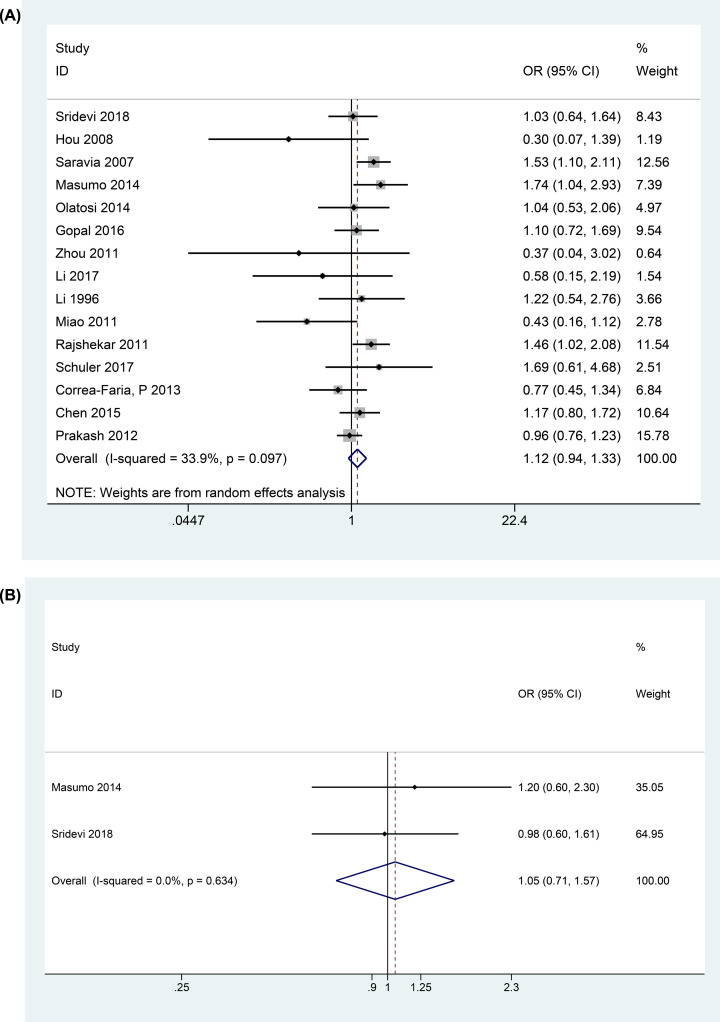
Forest plots showed the relationship between LBW and ECC (**A**) shows the meta-analysis resluts of ORs, (**B**) shows the meta-analysis results of the adjustment ORs.

The relationship between LBW and ECC is shown in Figure 3A. Heterogeneity across studies was *P*_heterogeneity_ = 0.097, *I^2^*= 33.9%. Meta-analysis of the included studies using random-effect model indicated that LBW could not predispose preschool children to ECC (OR = 1.12, 95% CI: 0.94–1.33, *P*>0.5). In addition, the meta-analysis of two studies including the adjustment ORs showed the consistent results (OR = 1.05, 95% CI: 0.71–1.57, *P*=0.802, *P*_heterogeneity_ = 0.63, *I^2^* = 0%) (Figure 3B).

**Figure 4:**
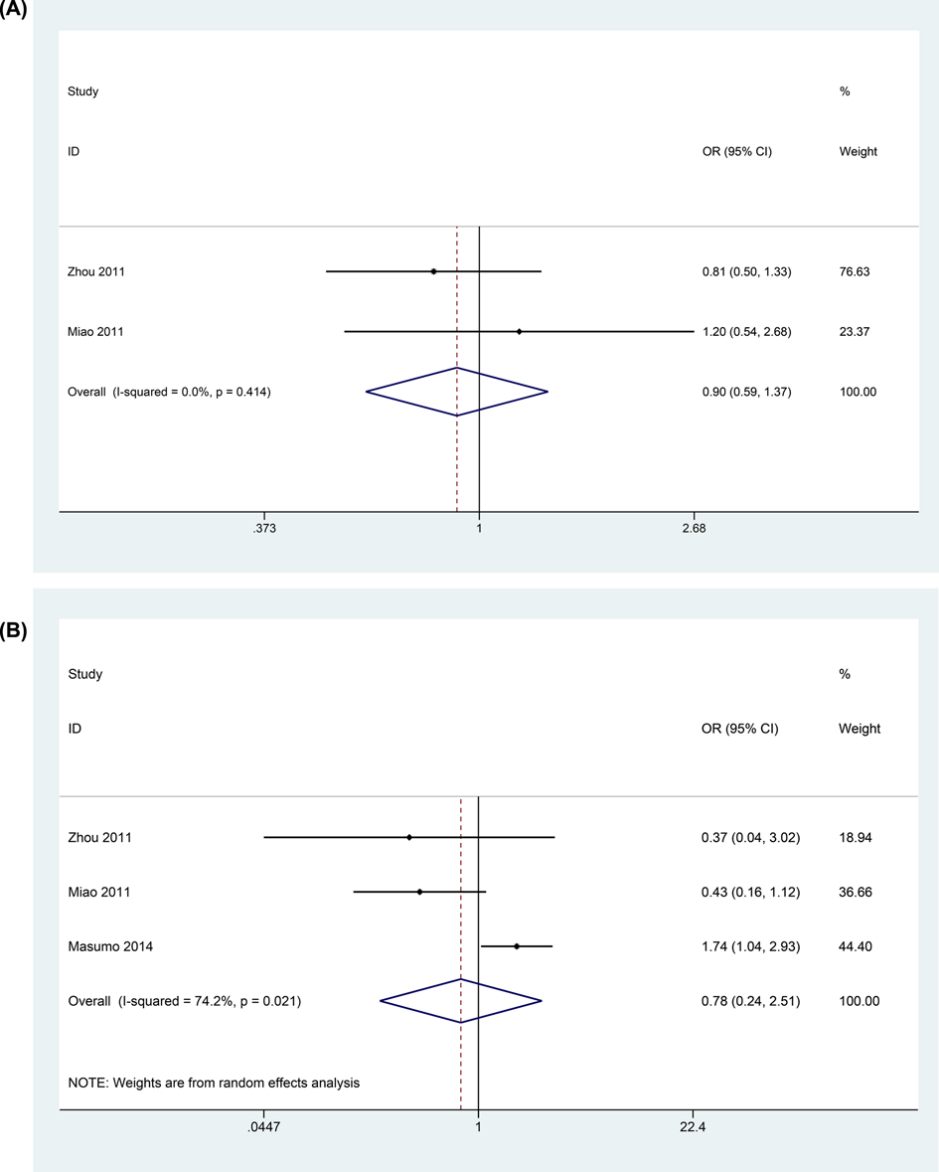
Forest plots showed the relationship between preterm, low birthweight and ECC when children were younger or equal 3 years old (online only)

As shown in Figure 4A, two studies investigated the relationship between preterm and ECC, the participants of which were younger or equal to 3 years old. Heterogeneity across studies was *P*_heterogeneity_ = 0.414, *I^2^*= 0%. Meta-analysis of the included studies using fixed-effect model showed that when children were younger or equal to 3 years old, preterm was not a risk factor for ECC (OR = 0.90, 95% CI: 0.59–1.37); Figure 4B showed the relationship between LBW and ECC when children were younger or equal to 3 years old. The meta-analysis results of these three studies indicated that LBW was not a risk factor for ECC (OR = 0.78, 95% CI: 0.24–2.51, *P*_heterogeneity_ = 0.021, *I^2^*= 74.2%).

### Sensitivity analysis

Sensitivity analysis was conducted by omitting one study at each time and recalculating the pooled results. Among the studies about premature and the risk of ECC, the overall risk estimation did not vary materially with a range from 1.54 (95% CI: 1.31–1.80) to 1.66 (95% CI: 1.43–1.93). The sensitivity analysis results of the relationship between LBW and ECC showed that the risk estimation range slightly changed from 1.07 (95% CI: 0.90–1.28) to 1.16 (95% CI: 0.98–1.35), indicating that the results were stable.

### Publication bias

As suggested by the Peters' test, publication bias was not found in the included studies(preterm and ECC: *P* = 0.993>0.1; LBW and ECC: *P* = 0.254>0.1).

## Discussion

A lot of studies suggested the relationship between preterm or LBW and ECC with an inconsistent result. There were many factors including different study methods, ages at examination, diagnosing criteria, which resulted in the contradiction. This meta-analysis was the first systematic evaluation of the relationship between preterm, LBW and ECC, published up to December 2019, with 25,166 participants from 22 epidemiological studies. The results of this meta-analysis not only further clarify the pathogenesis of ECC, but also provided a comprehensive guidance to prevent and block the progression of ECC.

In this meta-analysis, a total of 16 studies explored the relationship between preterm and ECC, which included 7 case–control studies and 9 cross-sectional studies with 21,279 participants. The results demonstrated that compared with full-term infants, preterm significantly increased the risk of ECC. Previous studies had explained the mechanisms.

Maternal health influenced the children’s birth conditions [41]. Alexander suggested that abnormal events in gestation such as smoking, drinking, metabolic disturbance, malnutrition and infection retarded infant growth, thus gave an adverse birth outcome [41]. Previous studies showed that when mothers experienced smoking exposure or alcohol, the development of primary teeth would be affected and consequently increased the caries incidence [42]. When pregnant women were metabolic disturbance or malnourished, the sufficient necessary nutrition could not be transported to fetus via the placenta [43], particularly the primary incisors mineralization started at the 14th gestational weeks and posterior teeth mineralized completely by 1 year after birth. Premature had an increased prevalence of EHP due to intrauterine undernutrition results from deficiency of vitamin A, C, D, calcium and phosphate [44], which were essential elements in teeth mineralization.

Numerous studies had reported that children with adverse birth outcomes were vulnerable to diseases because of the low immunity [45]. Fetal growth retardation resulted in deficits in cell-mediated immunity that persisted for years [46]. Caufield pointed out that there was a window of infectivity for initial acquisition of streptococcus mutans which was closely related to dental caries between 1 and 2 years of ages [46]. A grouping number of evidences indicating that preterm children with impaired immune system were more susceptible to infect streptococcus mutans due to the window of infectivity persist as long as 5 years [47]. In addition, the rough surfaces of demineralized deciduous teeth like a base for cariogenic bacteria to adhere and colonize easily that led to quick progression of dental caries [15]. Previous studies had established that adequate saliva, normal salivary flow rate and composition changed oral cavity microecological environment and the IgA prevented oral microbes from adhering to oral mucosa and teeth, which decreased the risk of dental caries [48]. Rythen et al. reported in a case–control study that premature had smaller secretions of stimulated saliva than full-term children [49], and Nogueira et al. found the level of salivary IgA in premature was 2.5-fold lower compared with that of full-term children [50].

Preterm children had difficulties in sucking, and they started bottle-feeding earlier and the duration was longer [51]. Breast milk was rich in nutrition that fermentable milk was hard to be replaced [52]. In addition, the substitutes of breast milk such as cow’s milk contained much higher amounts of sugar, which was cariogenic agent, predisposed infants to dental caries [52]. Premature infants were vulnerable to many serious sicknesses, such as respiratory disease or cardiovascular disease, because they missed the period for organ maturation [45]. Disturbed motor functions, cognitive and behavioral impairments led to a failure control of their oral conditions.

A total of 15 studies explored the relationship between LBW and ECC, including 6 case–control studies and 9 cross-sectional studies in this meta-analysis. The results suggested that LBW was not a risk factor for ECC. Masumo reported that LBW was closely related to ECC, but the relationship did not maintain significance after taking enamel defects, oral hygiene and high consumption of sugar into consideration [25]. Rajshekar detected a significant difference in caries prevalence between preterm low birth weight (PTLBW) infants and full-term normal birth weight (FTNBW) children. Because the case group children were PTLBW preschool children, it was difficult to eliminate the interference of preterm and investigate the relationship between LBW and ECC [30].

This meta-analysis systematically assessed the relationship between LBW and ECC by including 15 studies conforming to inclusion criteria and the results showed that LBW was not a risk factor for ECC. In 1985, Kramer concluded that there were 43 determinants of LBW, which were categorized as preterm and IUGR [9]. Nearly one-third of the LBW infants were full-term children. Saraiva explored the interaction between LBW and preterm and the investigation found that compared with PTLBW, children born with low weight and at full-term gestation ages were less likely to get caries [23]. In addition, LBW was one of the characteristics of the IUGR [9]. Birth weight as well as length and head circumference were crude measures of fetal growth. It could be hypothesized that IUGR fetuses presented more mature enamel development in comparison with preterm children, and normal enamel provided a structure foundation to inhibit the dental caries [23]. Furthermore, the increased utilization of antibiotic among growth-retarded children was thought to reduce the colonization of cariogenic bacteria [53].

The meta-analysis results indicated that when children were younger or equal to 3 years old, preterm and LBW were not risk factors for ECC. The main reason for the results was that the primary teeth of preterm or LBW children erupted later than normal infants. Backstorm et al. reported that the primary teeth of nearly 60% preterm or LBW children would erupt at 10 months after birth [54], and Sajjadian also revealed that there was a remarkable negative correlation between the eruption time of the first primary teeth and the birth weight [55]. It could be concluded that the adverse birth outcomes such as preterm or LBW did not predispose children to get caries when they were younger than 3 years old. This result was consistent with Nirunsittirat’s study. He suggested that this relation was because of the delayed teeth eruption and the short progressing time [5]. With the colonization and reproduction of cariogenic bacteria, the relationship between preterm or LBW and ECC became explicit. Due to the limited number of literatures conforming to the inclusion criteria, more studies need to be included for further study. The results also indicated that the relationship between preterm, LBW and ECC would be more precise if the study group participants were in different age ranges.

The quality of this meta-analysis was high and the results were scientific and reliable. The results of *Q*-test showed that the heterogeneity of the included studies was moderate. There were some factors influenced the heterogeneity potentially. Various ways to diagnose caries, such as visual examination, probing or combined with radiography were potential factors. Furthermore, the sensitivity analysis also showed that the results were stable and the Peters’ tests revealed no publication bias, which increased the strength of the study. All the included studies with a high or moderate quality indicating the meta-analysis results were real and reliable.

There were several potential limitations in the present study that warranted consideration. The classifications of birth weight were complex. The most cross-sectional studies divided it into two classes (>2500 g and <2500 g), while the others involved in fetal macrosomia (>4000 g), or VLBW (<1500 g). A grouping number of evidences indicated that the most of the VLBW infants were premature and they were probably in high risk of ECC [18]. The present study adopted the widely used classification that divided birth weight into two categories, which did not further investigate the relationship between VLBW and ECC. Further study should be focused on the relationship between VLBW and ECC. Additionally, some data about birth conditions were recorded in the birth certifications and reviewed by examiners. In many studies, the data were provided by parents or guardians. The memory bias generated information bias and might influence the accuracy. In addition, the languages of the included studies were only English and Chinese, and some studies in other languages that met the inclusion criteria might be lost which that increase the bias.

This meta-analysis indicated that preterm positively increased the risk of ECC; however, LBW was not. The results suggested that the preventive and blocking therapy for ECC should be the first choice. Much more attention should be paid to the health care for pregnant women during their perinatal periods and measures should be taken to reduce the prevalence of preterm. The clinical effectiveness would be much better if corresponding measures were taken to against ECC as soon as preterm infants just born.

## Abbreviations

AAPD: American Academy of Pediatric Dentistry
CI: confidence interval
ECC: early childhood caries
EHP: enamel hypoplasia
FTNBW: full-term normal birthweight
IUGR: intrauterine growth restriction
LBW: low birth weight
OR: odds ratio
PICO: Participants, Interference, Control, Outcome
PRISMA: Preferred Reporting Items for Systematic Reviews and Meta-analyses
PTLBW: preterm low birth weight
VLBW: very low birth weight
WHO: World Health Organization
